# EXOSC5 maintains cancer stem cell activity in endometrial cancer by regulating the NTN4/integrin β1 signalling axis

**DOI:** 10.7150/ijbs.86275

**Published:** 2024-01-01

**Authors:** Yu-Hao Huang, Wen-Ling Wang, Po-Hui Wang, Hsueh-Te Lee, Wen-Wei Chang

**Affiliations:** 1 Program in Molecular Medicine, National Yang Ming Chiao Tung University and Academia Sinica, Taipei 112304, Taiwan.; 2 Department of Biomedical Sciences, Chung Shan Medical University, Taichung 402306, Taiwan.; 3 Institute of Medicine, Chung Shan Medical University, Taichung 402306, Taiwan.; 4 Department of Obstetrics and Gynecology, Chung Shan Medical University Hospital, Taichung 402306, Taiwan.; 5 School of Medicine, Chung Shan Medical University, Taichung 402306, Taiwan.; 6 Institute of Anatomy & Cell Biology, College of Medicine, National Yang Ming Chiao Tung University, Taipei 112304, Taiwan.; 7 Taiwan International Graduate Program in Molecular Medicine, National Yang Ming Chiao Tung University and Academia Sinica, Taipei 115024, Taiwan.; 8 Brain Research Center, National Yang Ming Chiao Tung University, Taipei 112304, Taiwan.; 9 Department of Medical Research, Chung Shan Medical University Hospital, Taichung 402306, Taiwan.

**Keywords:** Endometrial carcinoma, cancer stem cells, c-MYC, EXOSC5, NTN4, integrin β1

## Abstract

Endometrial carcinoma (EC) is a common type of uterine cancer in developed countries, originating from the uterine epithelium. The incidence rate of EC in Taiwan has doubled from 2005. Cancer stem cells (CSCs) are a subpopulation of cancer cells that have high tumorigenicity and play a crucial role in the malignant processes of cancer. Targeting molecules associated with CSCs is essential for effective cancer treatments. This study delves into the role of Exosome component 5 (EXOSC5) in EC. Data from The Cancer Genome Atlas suggests a correlation between high EXOSC5 mRNA expression and unfavorable EC prognosis. EXOSC5 knockdown diminished EC-CSC self-renewal and reduced expression of key cancer stemness proteins, including c-MYC and SOX2. Intriguingly, this knockdown significantly curtailed tumorigenicity and CSC frequency in EC tumor spheres. A mechanistic examination revealed a reduction in netrin4 (NTN4) levels in EXOSC5-depleted EC cells. Moreover, NTN4 treatment amplified EC cell CSC activity and, when secreted, NTN4 partnered with integrin β1, subsequently triggering the FAK/SRC axis to elevate c-MYC activity. A clear positive relation between EXOSC5 and NTN4 was evident in 93 EC tissues. In conclusion, EXOSC5 augments NTN4 expression, activating c-MYC via the integrin β1/FAK/SRC pathway, offering potential avenues for EC diagnosis and treatment.

## Introduction

Endometrial carcinoma (EC) arises from the layer of cells that form the lining (endometrium) of the uterus, and it is the most common cancer of the female reproductive system in Europe and North America 1. In Taiwan, the incidence rate of EC doubled from 2005 to 2016 2. ECs are classified into two clinicopathologic types: estrogen-dependent endometrioid adenocarcinomas (type I) and estrogen-independent uterine serous carcinomas and clear cell carcinomas (type II) 3. Type I ECs often exhibit PTEN loss and PI3KCA mutation 4, 5, whereas type II ECs typically show p53 mutation and HER-2 overexpression 6. The majority of EC cases (80%) belong to type I 7. The prognosis of patients with EC in early stage (stage I or II) is generally favorable, but the prognosis of advanced stage (stage III or IV) or recurrent EC is poor, with 5-year overall survival rates of approximately 15% 8. Therefore, identifying novel pathogenic genes that could be used for early detection and as therapeutic targets is necessary to improve the prognosis of women with advanced or recurrent EC.

In many malignancies, a stem cell-like subset of cancer cells has been identified and referred to as cancer stem cells (CSCs) 9. CSCs are believed to have the potential to initiate tumors and facilitate metastasis to distant sites 10. Numerous studies showed that CSCs contribute to drug resistance through epithelial-mesenchymal transition 11 and participate in tumor angiogenesis 12 and immune evasion within the tumor microenvironment (TME) 13. The pluripotent transcription factors c-MYC, OCT4, BMI1, and NANOG have been shown to regulate the maintenance of CSCs 14. In addition, several intracellular signaling pathways, including Notch 15, Wnt/β-catenin 16, PI3K/AKT 17, and integrins 18, have been identified as vital regulators of CSC activity. Targeting CSCs is considered an effective strategy for successful cancer treatment to prevent tumor relapse 19. In patients with EC, high aldehyde dehydrogenase (ALDH)1A1 expression is associated with poor survival, and tumor spheres derived from clinical EC specimens showed high ALDH activity 20. Furthermore, the targeting transcription factors of stemness, such as BMI1^21^ and NANOG 22, holds high potential for EC treatment, suggesting that developing an anti-CSC therapeutic approach is an efficient method to treat ECs.

The RNA exosome complex comprises three cap proteins (EXOSC1-3) and six core proteins (EXOSC4-9) 23. This complex, which has 3ʹ-5ʹ exoribonuclease activity 24 is involved in mRNA turnover and quality control, rRNA processing, and small noncoding RNA processing 25. In addition to its function in RNA degradation and processing, the components of the exosome complex have been found to be involved in carcinogenesis. For example, the expression of EXOSC2 can be enhanced via tRNAGluUUC to promote breast cancer metastasis 26. Depletion of EXOSC4 in various cancer types leads to the suppression of cell growth and invasion 27. EXOSC5, also known as RRP46 or CML28, is a noncatalytic subunit of the RNA exosome complex. Previous studies indicated that EXOSC5 is upregulated in various tumors and cell lines 28, 29. Recently, EXOSC5 was identified as a novel prognostic marker in colorectal cancers, and its oncogenic role was found to be associated with the activation of the ERK and AKT pathways 30. Although EXOSC5 is a potential prognostic factor and oncogene in numerous cancer types, its functional roles and molecular mechanisms in ECs are largely unknown.

In this study, the role of EXOSC5 in regulating EC-CSCs was investigated, including their self-renewal capability and *in vivo* tumorigenicity. Furthermore, the underlying molecular mechanisms of EXOSC5 in regulating CSC activity in EC were explored.

## Materials and Methods

### Cell lines

EC cell lines, including AN3CA, HEC1A, HEC1B, KLE, RL95-2, Ishikawa, and HEC59, were procured from the American Type Culture Collection (ATCC) (Manassas, VA, USA). They were cultured in accordance with the recommendations provided by ATCC. The identity of these cell lines was ascertained through STR analysis, conducted by the Center for Genomic Medicine at National Cheng Kung University (Tainan, Taiwan).

### Establishment of primary EC cells from Taiwanese endometrial cancer tissues

The collection of EC specimens received approval from the Institutional Review Boards at Chung Medical University Hospital (Taichung, Taiwan), approval No. CS18180. Primary human EC cells were isolated from fresh EC surgical specimens sourced from Taiwanese female patients. This isolation was achieved via enzymatic dissociation with a collagenase/hyaluronidase solution (STEMCELL Technologies Inc., Vancouver, BC, Canada), followed by tumoursphere cultivation. The subsequently formed secondary tumourspheres were dissociated into single-cell suspensions (dubbed EMC) using the HyQTase solution (HyClone Laboratories Inc., Logan, UT, USA) and cultured at 37°C in a 5% CO2 atmosphere using DMEM augmented with 10% fetal bovine serum (HyClone Laboratories Inc.).

### Tumoursphere cultivation

EC cells, once harvested from monolayer culture, were adjusted to a density of 1x10^4^ cells/ml in DMEM/F12 medium. This medium was enhanced with the following supplements: 1% methylcellulose (Sigma-Aldrich, St. Louis, MO, USA), 0.4% bovine serum albumin (Gibco Inc., Waltham, MA, USA), 10 ng/ml EGF (PeproTech, Rocky Hill, CA, USA), 10 ng/ml bFGF (PeproTech), 5 µg/ml insulin (Sigma-Aldrich), 1 µM hydrocortisone (Sigma-Aldrich), 0.5X B27 supplement (Gibco), and 4 µg/ml heparin (Sigma-Aldrich). 2 ml of this cell suspension was added to the wells of an ultralow attachment 6-well plate (Corning Corporate, Corning, NY, USA) and incubated for a period spanning 7-10 days at 37°C and 5% CO_2_. For secondary tumorsphere formation, primary tumorspheres were collected using a 100 µm cell strainer (BD Biosciences, Franklin Lakes, NJ, USA), disaggregated into single-cell suspensions with HyQTase (Merck Millipore, Burlington, MA, USA), and plated into ultralow attachment plates at a density of 5x10^3^ cells/ml.

### Knockdown of EXOSC5 by lentiviral transduction of shRNA

The plasmids pCMVΔ8.91, pMD.G, and gene-specific shRNA (EXOSC5#1: TRCN0000286812; EXOSC5#2: TRCN0000306868; LacZ: TRCN0000231722) were procured from the National RNAi Core Facility (Academia Sinica, Taipei, Taiwan). Lentiviruses were generated by cotransfecting HEK-293T cells with a plasmid DNA mixture: shRNA (2.5 μg), pCMVΔ8.91 (2.25 μg), and pMD.G (0.25 μg) using the NTRII DNA transfection reagent (T-Pro Biotechnology, New Taipei City, Taiwan. Catalog No. #JT97-N002M). At 48 h post-transfection, media containing lentiviruses were harvested, filtered through a 0.45 μm filter, and used for transduction when cells reached 30% confluence. This was done in the presence of 8 μg/mL polybrene for 24 h. Subsequently, the medium was replaced with a fresh one, supplemented with 2 μg/mL puromycin, to select successfully transduced cells.

### Western blotting and coimmunoprecipitation (Co-IP)

Western blot analysis was conducted using whole-cell protein lysates obtained with the NETN lysis buffer (100 mM NaCl, 20 mM Tris-Cl (pH 8.0), 0.5 mM EDTA, 0.5% (v/v) NP-40). Protein concentrations were determined utilizing the Pierce™ BCA Protein Assay Kit (Thermo Fisher Scientific Inc., Waltham, MA, USA). Thirty micrograms of total protein underwent separation by SDS‒PAGE and was then transferred onto a PVDF membrane. This membrane was incubated overnight at 4 °C with primary antibodies targeted against specific proteins, using 5% BSA/TBS-T buffer with gentle shaking. Horseradish peroxidase (HRP)-conjugated secondary antibodies detected the primary antibodies, and the resulting signals were developed via a chemiluminescent reagent (Amersham ECL Prime Western blotting Detection Reagent, GE Healthcare Life Sciences, Chicago, IL, USA). Images were captured with the Luminescence-Image Analyzer (Amersham™ Imager 680, GE Healthcare Life Sciences). For co-IP, 1 mg of total cellular proteins was combined with 2 μg of a specific antibody or normal rabbit IgG in IP buffer and left overnight at 4 °C. The immune complexes were then precipitated by protein G Mag Sepharose beads (GE Healthcare Life Sciences) at 4 °C for 2 h. After washing three times with IP buffer, the precipitated immune complexes were eluted in 2X sample buffer and separated via 10% SDS‒PAGE. They were then subjected to Western blot analysis as previously detailed.

### *In vivo* tumorigenicity and limiting dilution assay

Five-week-old female NOD/SCID mice, procured from Lesco Biotechnology (Taipei, Taiwan), were employed to assess the *in vivo* tumorigenicity of tumoursphere cells derived from AN3CA cells. Following dissociation with Accutase (Gibco), a single-cell suspension of primary tumourspheres from AN3CA cells was prepared and subsequently transduced with either sh-LacZ or sh-EXOSC5 lentivirus. These cells were subcutaneously injected into the interscapular area of NOD/SCID mice at varying cell concentrations: 1x10^5^ cells/site, 2x10^4^ cells/site, and 1x10^3^ cells/site for the sh-LacZ group, and 5x10^5^ cells/site, 1x10^5^ cells/site, and 2x10^4^ cells/site for the sh-EXOSC5 group. Tumour formation was monitored biweekly, with tumour volume determined using the formula: D × d^2/2 (D represents length, while d represents width).

### Immunofluorescence (IF) analysis

Cells were fixed with 4% paraformaldehyde, permeabilized with PBS containing 0.1% Triton X-100, blocked with 1% BSA and incubated with primary antibodies at 4ºC overnight. After washing with PBS, fluorescein-conjugated secondary antibodies were added, and fluorescent images were captured under a ZEISS LSM 510 Meta confocal microscope (Oberkochen, Germany).

### Tissue microarray and immunohistochemistry (IHC) analysis

Human endometrial cancer tissue microarray slides containing 97 cases of EC patients and 5 normal uterus tissues were purchased from TissueArray.Com LLC (Derwood, MD, USA. Cat. No. EM1021c); for these microarrays, tissue collection was performed with informed consent from the donors. After dewaxing and antigen retrieval, the slides were incubated with each antibody, including anti-EXOSC5 (GeneTex Inc., Hsinchu City, Taiwan. Catalog No. GTX118473), anti-NTN4 (Sigma-Aldrich, Catalog No. HPA049832), anti-phospho-FAK^Tyr397^ (ABclonal, Inc., Woburn, MA, USA. Catalog No. AP0302), or anti-c-MYC (Santa Cruz Biotechnology Inc., Dallas, TX, USA. Catalog No. sc-40), followed by signal development with a standard avidin-biotin-peroxidase complex method with 3,3'-diaminobenzidine substrates (DAKO, Carpinteria, CA). Negative controls included serial sections from which either the primary or secondary antibodies were excluded. The slides were counterstained with haematoxylin and mounted with Permount (Merck, Darmstadt, Germany), and the images were captured and analysed with a TissueFAXS Plus Microscopy System (TissueGnostics GmbH, Vienna, Austria).

### Fluorescence-activated cell sorting (FACS)

Primary EMC cells were dissociated into single-cell suspensions using an enzyme-free cell dissociation buffer (Catalog No. 13151014, Gibco™, Thermo Fisher Scientific). They were then stained with an APC-conjugated mouse anti-human CD29 antibody (Catalog No. 559883, BD Biosciences) at room temperature for 30 minutes. Cells exhibiting high (CD29^High^) or low (CD29^Low^) levels of CD29 were sorted using a FACSAriaII cell sorter (BD Biosciences). The expression of CD29 on the sorted cells was subsequently analyzed using FlowJo software (version 10.8.0, FlowJo, LLC., USA).

### RNA sequencing

HEC1A cells were seeded onto 6 cm plates and transduced with either LacZ- or EXOSC5-specific shRNAs carrying lentiviruses for 24 hours. This was followed by a selection with 2 μg/mL puromycin for two days. Total RNA was extracted and purified with the Quick-RNA™ MiniPrep Kit (Catalog No. R1054, Zymo Research, Irvine, CA, USA). RNA sequencing was carried out by BIOTOOLS Biotech Co., Ltd. (Taipei, Taiwan). Raw reads underwent filtering by Trimmomatic, followed by expression normalization via RLE/TMM/FPKM methods. Differentially expressed genes, both upregulated and downregulated, were identified using a stringent fold change cut-off value of 1.5 and an adjusted p-value less than 0.05.

### Single-cell RNA sequencing

Primary EMC cells were prepared for single-cell RNA sequencing. The microbead-captured single-cell library was constructed using the BD Rhapsody™ Single-Cell Analysis System (Catalog No. 633701, BD Biosciences) in accordance with the manufacturer's instructions. Single cells underwent multiplex labeling with a unique 45-nucleotide barcode tag provided by the Human Single-Cell Multiplexing Kit based on antibody-oligo technology (Catalog No. 633781, BD Biosciences). These tag-labeled cells were pooled together in equal proportions and loaded into a single Rhapsody™ Cartridge (Catalog No. 633733, BD Biosciences). Following a wash, lysis buffer was added to lyse the cells, facilitating RNA molecule hybridization with the beads. These beads were subsequently harvested into a single tube, where double-stranded cDNA underwent synthesis through a series of steps: reverse transcription, second-strand synthesis, end preparation, adapter ligation, and transcriptome amplification. The cDNA library, produced using the Whole Transcriptome Analysis (WTA) Amplification Kit (Catalog No. 633801, BD Biosciences), was then forwarded to BIOTOOLS CO., LTD. (New Taipei City, Taiwan) for RNA sequencing. The resulting sequence data, represented as a matrix, detailed transcript counts per gene for each individual cell. This data matrix underwent clustering analysis and was then reduced for 2-dimensional visualization, producing uniform manifold approximation and projection (UMAP) plots.

### c-MYC reporter assay

A luciferase-based c-Myc reporter vector (pMyc-Luc) was acquired from Signosis Inc. (Catalog No. LR-2018, Signosis Inc., Santa Clara, CA, USA). It was combined with the pRL vector, which carries the Renilla luciferase gene, to calibrate the transfection efficiency. This mixture was then complexed with the TransIT-X2™ transfection reagent (Mirus Bio LLC., Madison, WI, USA) and left to incubate at room temperature for 30 minutes. After seeding the cells in 12-well plates at a density of 1x10^5 cells/well and allowing them to attach overnight, the DNA/transfection reagent complex was introduced to the cells. After 24 hours, the media was replaced with fresh culture media. Total cell lysates were subsequently collected using passive lysis buffer (Promega Corporation, Madison, WI, USA). Both the Firefly and Renilla luciferase activities were quantified using the Dual-Luciferase assay system (Promega) on a GloMax® 20/20 Luminometer (Promega).

### Statistical analysis

Values from qRT-PCR, tumourspheres, and relative light units (RLU) are presented as mean ± SD (standard deviation). All statistical evaluations were carried out using GraphPad Prism 5.0 (GraphPad, San Diego, CA, USA). Quantitative data were subjected to a Student's t-test for comparisons between two distinct groups. For comparisons involving more than two groups, the Tukey-Kramer post hoc test was utilized. The overall survival (OS) between the two groups was gauged using the Kaplan-Meier method complemented by a log-rank test. A p-value less than 0.05 was deemed statistically significant.

## Results

### EXOSC5 expression is correlated with poor clinical outcome in patients with EC

**Figure 1:**
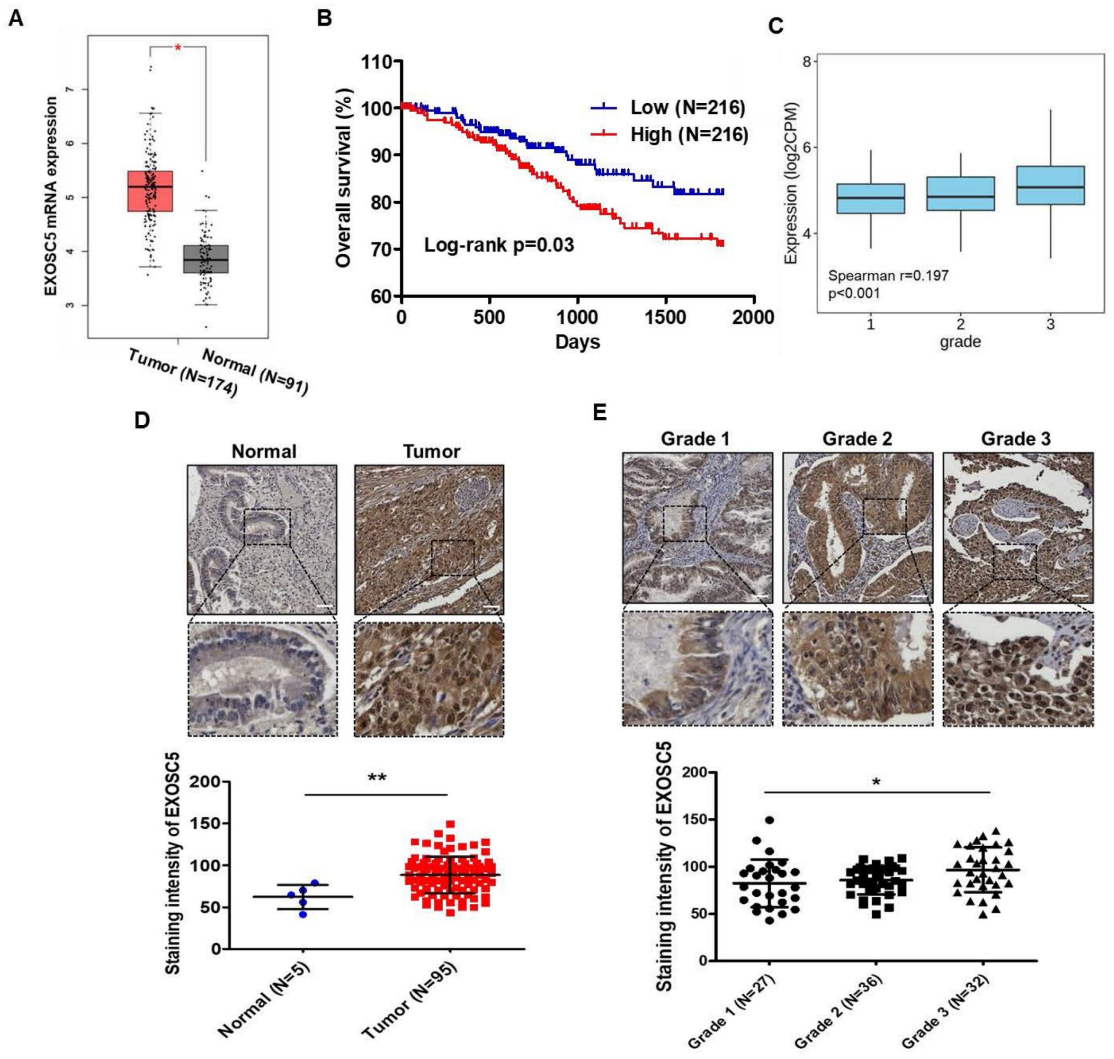
** The prognostic potential of EXOSC5 in ECs.** (A) Comparison of mRNA levels of EXOSC5 between normal endometrial tissues and EC specimens, sourced from the GEPIA2 website (http://gepia2.cancer-pku.cn/#index). *, p< 0.05 by Student's *t*-test. (B) Kaplan-Meier curve depicts overall survival (OS) of EC patients in the TCGA database, differentiated based on the median level of EXOSC5 mRNA expression among samples. Significance was assessed using the log-rank test. (C) Spearman correlation analysis between EXOSC5 and histology grade in TCGA EC specimens, visualized using the TISIDB web tool. (D) Protein expression of EXOSC5 in normal or EC tissues was ascertained using IHC staining on tissue microarray slides. The upper panel presents representative images of EXOSC5 staining intensity in normal uterine tissue or EC tumor tissue, with the dotted box highlighting a magnified view of the indicated area. The lower panel shows quantification data after analysis by HistoQuest software. Data is represented as mean ± SEM; **, p< 0.01 by Student's t-test. (E) The upper panel displays representative images of EXOSC5 staining intensity across different histologic grades of EC specimens, with the dotted box showcasing a magnified view of the indicated area. The lower panel presents the quantification data. Data is represented as mean ± SEM; *, p< 0.05 by Tukey's Multiple Comparison Test. Scale bars in (D) and (E) represented 50 µm in length.

Analysis of The Cancer Genome Atlas (TCGA) database by using GEPIA2 webtool showed that the level of EXOSC5 mRNA was significantly higher in EC tumor tissues than in normal endometrial tissues (Fig. 1A). A positive correlation was found between EXOSC5 mRNA expression and shortened survival in patients with EC (Fig. 1B). An evident correlation was also detected between EXOSC5 expression and tumor histological grade (grades 1-3) in EC specimens (Fig. 1C). However, no significant difference was noted concerning clinical stages (Fig. S1). IHC scoring showed a higher protein level of EXOSC5 among 95 EC tissues than among five normal uterus tissues. EXOSC5 protein was found to localize in the nucleus and cytosol of EC tissues (Fig. 1D). Moreover, EXOSC5 protein expression was higher in poorly differentiated EC tissues (grade 3 in Fig. 1E). These results indicated that EXOSC5 expression is positively associated with poor prognosis in patients with EC.

### EXOSC5 expression correlates with cancer stemness in EC cells

**Figure 2:**
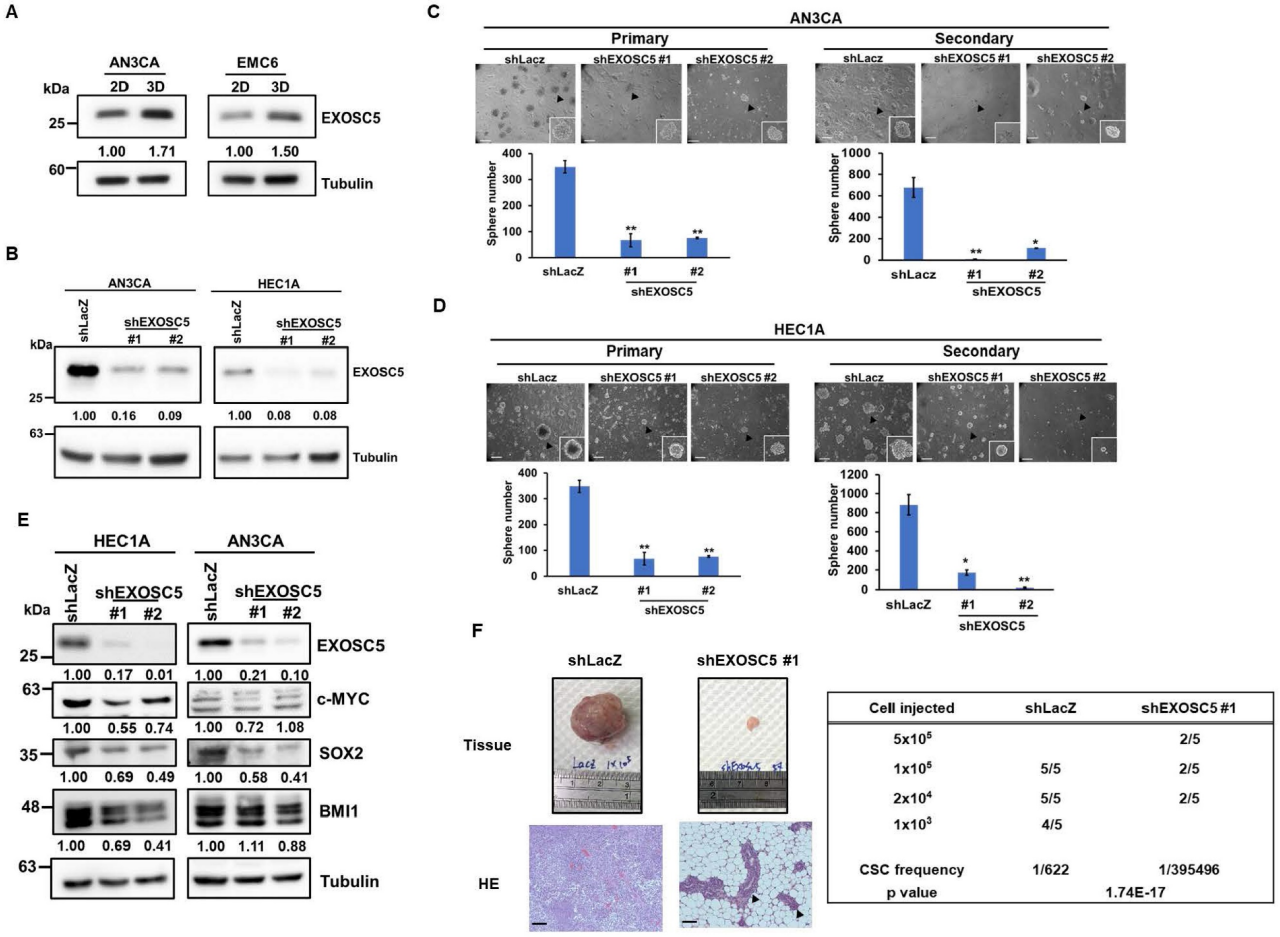
** EXOSC5 enhances CSC activity and self-renewal in ECs.** (A) Western blot detected the protein expression of EXOSC5 in adherence (2D) or tumorsphere (3D) cultured AN3CA and EMC6. (B) AN3CA or HEC1A cells were transduced with lentiviruses carrying either LacZ-specific (shLacZ) or EXOSC5-specific (shEXOSC5) shRNA. The efficiency of knockdown was determined by Western blot. (C, D) AN3CA cells (C) or HEC1A cells (D) transduced with shRNA were subjected to primary sphere cultivation, and the sphere numbers were counted on Day 7 (left). After counting, primary spheres were dissociated into a single-cell suspension using Accutase, followed by secondary sphere cultivation. The spheres were then counted on Day 7 post-seeding (right). Scale bars represented 100 µm in length. Data are depicted as mean ± SD. *, p< 0.05; **, p< 0.01; ***, p< 0.001 by Student's *t*-test. (E) After transduction with specific shRNA carrying lentiviruses (shLacZ for control, shEXOSC5#1, and shEXOSC5#2) for 72 hours, the protein expressions of EXOSC5, c-MYC, SOX2, and BMI1 in AN3CA and HEC1A cells were determined by Western blot. (F) The CSC frequencies in control (shLacZ) or EXOSC5-depleted (shEXOSC5#1) AN3CA cells were ascertained by assessing tumorigenicity in NOD/SCID mice using the indicated injection cell number. Frequencies were calculated using the ELDA software (https://bioinf.wehi.edu.au/software/elda/index.html).

Based on the TCGA EC dataset, EXOSC5 was found to be positively correlated with the surface CSC markers of EC 31, such as CD44 and CD133 (Figs. S2A and S2B). Pluripotent transcription factors, including c-MYC, OCT4, and BMI1, were also positively correlated with EXOSC5 in patients with EC (Figs. S2C-S2E). Tumor sphere culture methods were used on two EC cell lines (AN3CA and HEC1A) to enhance CSC subpopulations to further validate the enrichment of EXOSC5 protein expression in EC-CSCs. The results indicated an elevated level of EXOSC5 protein expression within the EC-CSCs (Fig. 2A). Lentiviral-based shRNA was used to knockdown the expression of EXOSC5 in AN3CA and HEC1A cells to characterize the functional role of EXOSC5 in EC cells (Fig. 2B). The analysis of tumor sphere formation revealed that the knockdown of EXOSC5 reduced the primary and secondary tumor sphere numbers in both EC cell lines (Figs. 2C and 2D), indicating that EXOSC5 is involved in the self-renewal capability of EC-CSCs. Moreover, downregulated cancer stemness proteins, including BMI1, c-MYC, and SOX2, were observed in EXOSC5 knockdown EC cells (Fig. 2E). In-vivo limiting dilution tumorigenicity assays were performed to reinforce the relationship between EXOSC5 expression and EC-CSCs. The EXOSC5-depleted EC cells presented significantly lower tumorigenicity than the control cells, with a significantly decreased CSC frequency (Fig. 2F). These results indicated that EXOSC5 plays an important role in regulating the stemness of EC.

### Netrin4 is a potential target of EXOSC5 in EC

**Figure 3:**
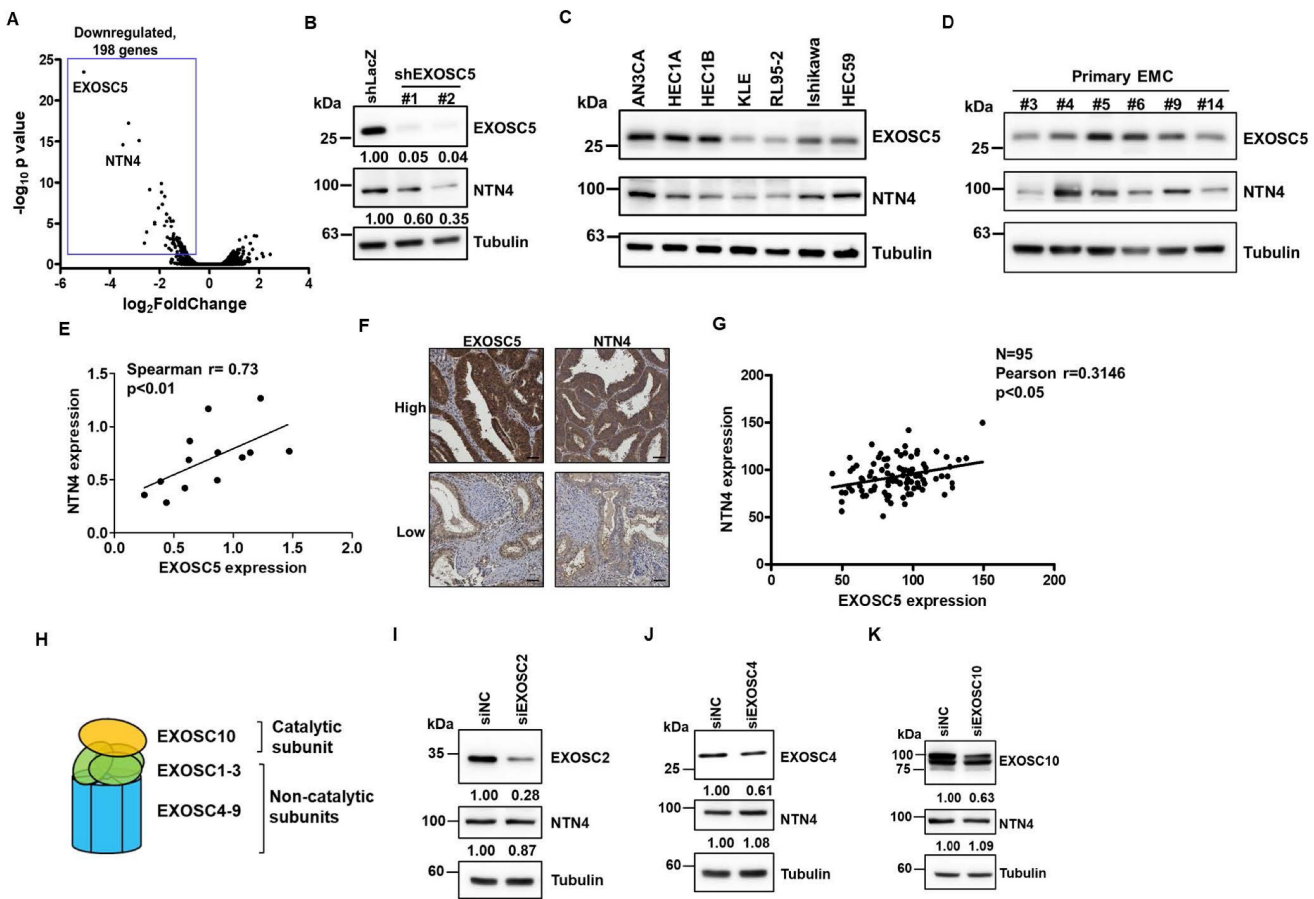
** Netrin4 is the potential target of EXOSC5 in EC.** (A) Volcano plot depicting differential gene expression between EXOSC5-knockdown and control HEC1A cells based on RNA sequencing analysis. (B) Western blot detection of EXOSC5 and NTN4 protein expressions in HEC1A cells post EXOSC5 knockdown (shEXOSC5#1 or shEXOSC5#2). (C, D, E) Protein expressions of EXOSC5 and NTN4 in multiple EC cell lines (C) and primary EC cells (D) determined by Western blot. Spearman's correlation analysis was used to assess the correlation between EXOSC5 and NTN4 expressions. (F, G) EXOSC5 and NTN4 protein expressions in EC tissue microarray slides were analysed via IHC. Representative images of high or low expression levels of EXOSC5 and NTN4 staining intensities are presented in (F). Pearson's correlation analysis was employed to evaluate the relationship between EXOSC5 and NTN4 expressions (G). Scale bars in (F) represented a length of 50µm. (H) Schematic representation of RNA exosome subunit assembly. (I, J, K) AN3CA cells, transfected with specific siRNA targeting EXOSC2 (I), EXOSC4 (J), or EXOSC10 (K), were harvested for protein analysis via Western blot. siNC represents negative control siRNA.

The RNA alterations in EXOSC5-depleted HEC1A cells compared with those in control cells were analyzed via RNA sequencing (RNA-seq) to explore the potential downstream targets of EXOSC5. Notably, the limited upregulation of genes could be found in EXOSC5 knockdown HEC1A cells (Fig. 3A) suggests that EXOSC5 might not majorly influence mRNA degradation in EC cells, hinting at an RNA exosome-independent activity for EXOSC5. Moreover, netrin4 (NTN4) was the most significantly downregulated gene in EXOSC5-depleted HEC1A cells versus control cells (Fig. 3A). The mRNA and protein expression levels of NTN4 were confirmed by qRT-PCR (Figs. S3 and S4) and Western blot (Fig. 3B). Numerous EC cell lines and primary EC cells were collected from Taiwanese patients to strengthen the correlation between the expression of EXOSC5 and NTN4 in EC. The results of Western blot showed that EXOSC5 expression was significantly positively correlated with NTN4 expression in EC cell lines and primary EC cells (Figs. 3C-3E). Moreover, the expression of EXOSC5 and NTN4 in tissue microarray slides containing 95 EC specimens was analyzed by IHC staining. A significant positive correlation was observed between the protein expression of EXOSC5 and NTN4 in EC tissues (Figs. 3F and 3G). The expression of subunits in the RNA exosome complex, including the catalytic subunit of EXOSC10 and two noncatalytic subunits of EXOSC2 and EXOSC4 (Fig. 3H), was further inhibited with RNA interference to clarify the relationship between the RNA exosome function of EXOSC5 and its regulation of NTN4 performance. The results showed that the depletion of EXOSC2 (Fig. 3I), EXOSC4 (Fig. 3J), or EXOSC10 (Fig. 3K) did not cause any significant alteration in NTN4 expression. Thus, NTN4 appears to be a potential target of EXOSC5 through RNA exosome-independent mechanism in EC cells.

### EXOSC5 enhances cancer stemness via NTN4/integrin β1 pathway

**Figure 4:**
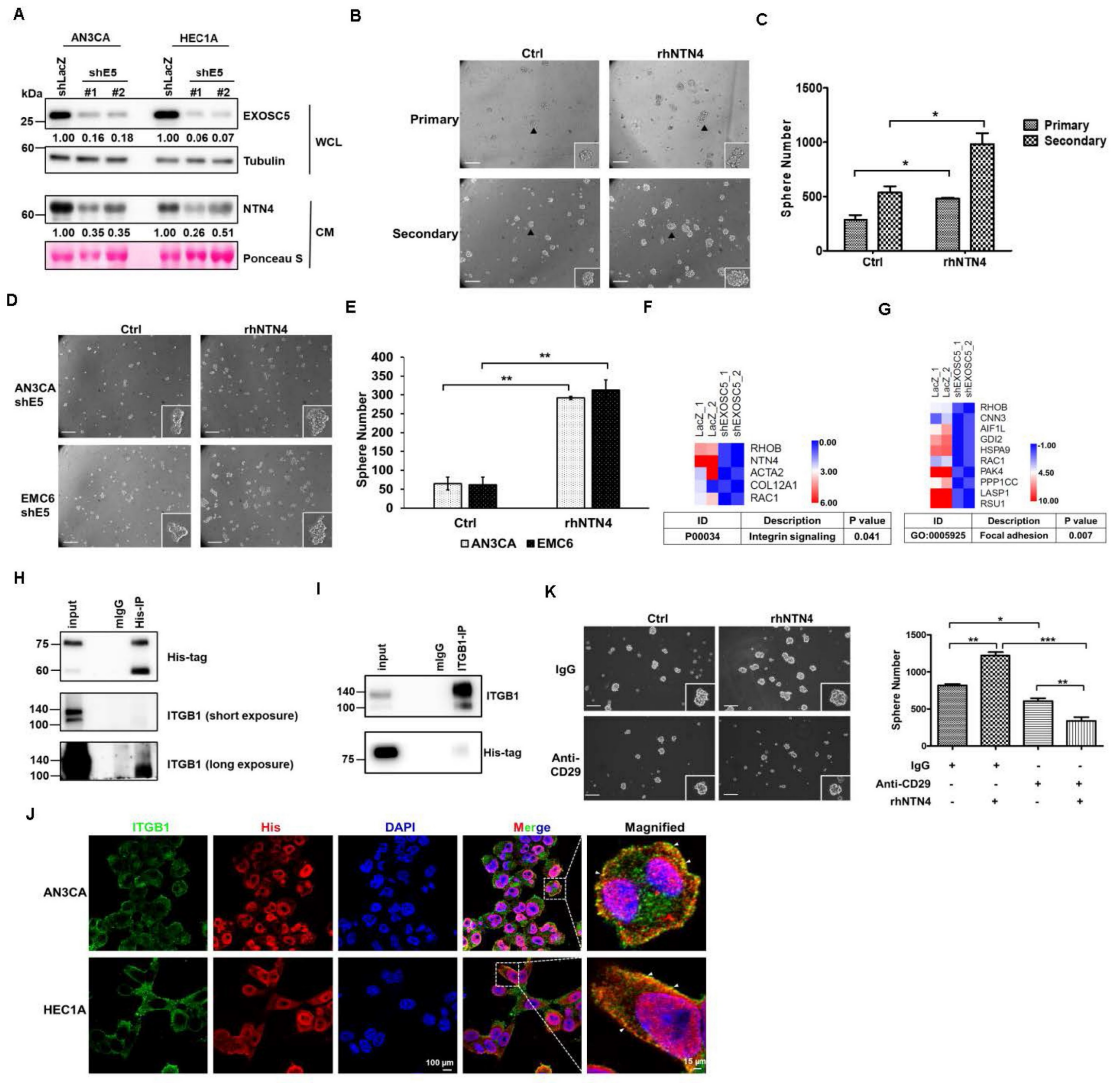
** EXOSC5 increases CSCs activity via NTN4/integrin β1 pathway.** (A) Levels of secreted NTN4 from conditional medium (CM) of EXOSC5-knockdown EC cells (shE5#1 or shE5#2) were detected by Western blot. WCL stands for whole cell lysate. (B, C) The effect of recombinant NTN4 (rNTN4) on CSC activity in AN3CA cells was assessed using a tumorsphere assay. Representative images of primary and secondary tumorspheres are shown in (B). Tumorsphere count quantification is presented in (C) with data shown as mean ± SD. Scale bars represent 100µm. *, p< 0.05 as determined by Student's *t*-test. (D, E) The influence of rNTN4 on CSC activity in EXOSC5-knockdown AN3CA cells was evaluated through a tumorsphere assay. Representative images of tumorspheres are shown in (D). Quantification data is presented in (E) as mean ± SD. **, p< 0.01 by Student's *t*-test. Scale bars indicated 100µm in length. (F-G) Heatmaps showcasing gene expression associated with the integrin signal (F) and focal adhesion (G) in EXOSC5-knockdown HEC1A cells compared to shLacZ-transduced cells were presented. (H-I) Whole cell lysates from AN3CA cells, treated with 150ng/ml rhNTN4 for 1 hour, were subjected to immunoprecipitation using anti-His (H) or anti-ITGB1 (I) antibodies. Western blot analysis was used to detect rhNTN4 and integrin β1 in the precipitated proteins. (J) AN3CA cells, treated with 150 ng/ml rhNTN4 for 1 hour, were fixed and subjected to immunofluorescent staining with anti-His and anti-ITGB1 antibodies. White arrows in the magnified images point to colocalized signals. (K) AN3CA cells, treated with 50 ng/ml rNTN4 in the presence or absence of the anti-CD29 neutralization antibody, had their CSC activity assessed using a tumorsphere assay. Representative tumorsphere images are displayed on the left, and quantification data is on the right, presented as mean ± SD. Statistical significance is indicated as *, p< 0.05; **, p< 0.01; ***, p< 0.001, determined by Tukey's Multiple Comparison Test.

NTN4, also known as β-netrin, is a secretory netrin described to be a component located in the basement membranes of the vasculature of the kidney and ovaries 32. NTN4 has also been demonstrated to act as a regulator of proliferation and metastasis in cancers 33, 34. Conditioned medium (CM) derived from EXOSC5-depleted EC cells was collected to evaluate whether EXOSC5 could affect the secretion of NTN4 in EC cells. The CM derived from EXOSC5-depleted EC cells showed significantly lower levels of secreted NTN4 than the CM from control cells (Fig. 4A). Previous studies showed that secreted NTN4 can promote proliferation and angiogenesis in endothelial cells 36. After microvascular endothelial cells were treated with CM derived from EXOSC5-depleted (shEXOSC5-CM) or control (shLacZ-CM) EC cells, the observational results showed that shEXOSC5-CM may impair tube formation in endothelial cells (Fig. S5). Recombinant human NTN4 protein (rhNTN4) was used to treat EC cells, and then the tumor-sphere formation capability was analyzed to clarify the function of secreted NTN4 in regulating cancer stemness. According to primary and secondary spheroid cultures, rhNTN4 enhanced CSC activity and self-renewal capability in EC cells (Figs. 4B and C). The spheroid formation capability of EXOSC5-depleted EC cells treated with rhNTN4 was analyzed to examine whether EXOSC5 promotes cancer stemness through secreted NTN4. Indeed, secreted NTN4 restored the CSC activity of EXOSC5-depleted EC cells (Figs. 4D and E). Overall, the results showed that EXOSC5 maintains cancer stemness via regulation of NTN4 secretion.

Next, Gene Ontology (GO) and pathway analysis (PANTHER and KEGG) were utilized to analyze the RNA-seq data of EXOSC5-depleted EC cells to elucidate the pathways by which EXOSC5/NTN4 modulates EC-CSC features. The differentially expressed genes related to EXOSC5 were enriched in integrin signaling (Fig. 4F) and focal adhesion (Figs. 4G and S6) in EC cells. Previous studies indicated that NTN4 is a ligand of integrin α6β1 that activates integrin downstream signaling in mouse neural stem cells 35 and endothelial cells 36. Integrin β1 is also the critical regulator of CSCs in various cancers 37, 38. Therefore, whether NTN4 is the ligand of integrin β1 in EC was investigated. The results of co-immunoprecipitation (IP) and immunofluorescence (IF) analysis revealed that secreted NTN4 interacted with integrin β1 in EC cells (Figs. 4H-4J). EC cells were treated with an integrin β1 blockade antibody (anti-CD29) or isotype IgG followed by rhNTN4 treatment to determine whether secreted NTN4 promotes CSC activity by modulating the integrin β1 pathway. The results demonstrated that anti-CD29 treatment significantly reduced the CSC activity of rhNTN4-treated EC cells (Fig. 4K). Together, these data suggested that secreted NTN4, which is the ligand of integrin β1, enhances cancer stemness by regulating integrin β1 signaling.

### Integrin β1 is a surface marker for enrichment of EC-CSCs

**Figure 5:**
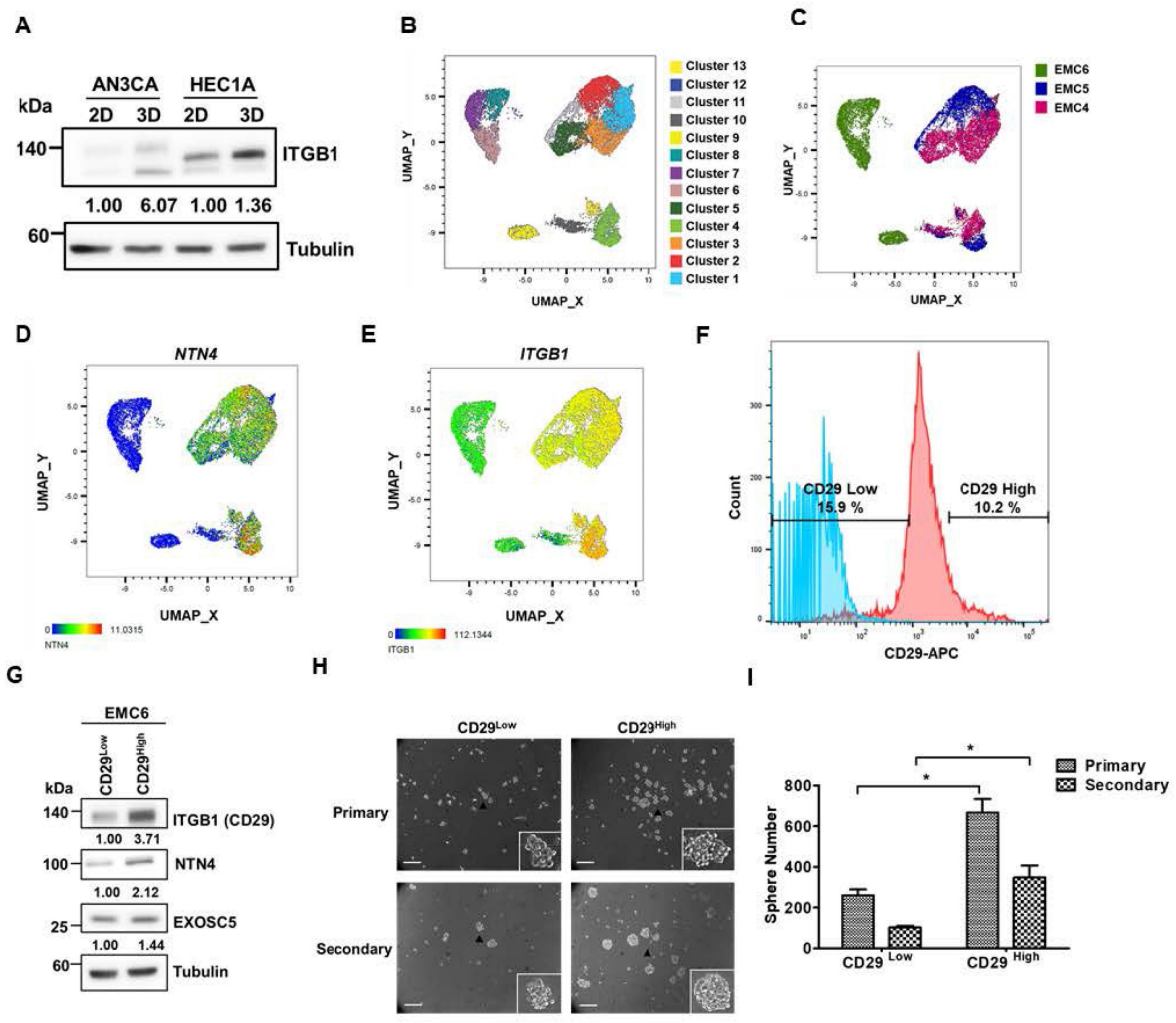
** Integrin β1 is an enrichment marker for EC-CSCs.** (A) Protein expression of integrin β1 in adherent (2D) and tumorsphere (3D) cultured AN3CA and HEC1A cells was analyzed using Western blot. (B, C) The combined single cell RNA sequencing (scRNA-Seq) data of three primary EC cell samples (EMC4, EMC5, and EMC6) were visualized using an UMAP plot to identify distinct cell clusters (B). Cells from each primary EC sample are distinguished by different colours and are presented in (C). (D-E) UMAP plots of the scRNA-Seq data from EC cells display the mRNA expression levels of NTN4 (D) and ITGB1 (E). (F) Representative populations of CD29^High^ and CD29^Low^ within EMC6 cells (highlighted in red) were identified using flow cytometry. The blue colour represents the unstained sample of EMC6 cells. (G) Protein levels of integrin β1, NTN4, and EXOSC5 in the CD29^High^ and CD29^Low^ populations of EMC6 cells were analysed using Western blot. (H, I) The CSC activity of CD29^High^ and CD29^Low^ cells derived from EMC6 was assessed via a tumorsphere assay. Representative images of primary and secondary tumorspheres are shown in (H). Quantification of the tumorsphere numbers is presented in (I) as mean ± SD. *, p< 0.05 as determined by Student's *t* test. Scale bars indicated 100 µm in length.

Integrin β1, also known as CD29, has been identified as a CSC marker and therapeutic target in colon and oral cancer 39, 40. The results of spheroid culture exhibited that integrin β1 was enriched in the CSC populations of EC cells (Fig. 5A). Three primary EC cell lines (CSC enriched by spheroid culture) were used to perform single-cell RNA-seq to hypothesize that integrin β1 is a potential target for EC-CSC treatment. The results of clustering analysis revealed that NTN4 expression was highly correlated with integrin β1 expression (Figs. 5B-5E). Integrin β1 high/low cancer cells were sorted from primary EC cells, and cancer stemness was assessed using spheroid formation assay to evaluate the importance of surface integrin β1 in EC-CSCs. Integrin β1 high/low populations were sorted from EMC6 cells because EMC4 and EMC5 cells possess high endogenous integrin β1 expression in most cells (Figs. S7A and S7B). The results showed that the integrin β1 high population had high NTN4 expression (Fig. 5G) and higher CSC activity and self-renewal capability than other primary EC cells (Fig. 5H). These results suggested that integrin β1 is an enrichment marker and a potential target for CSCs in EC.

### Secreted Netrin4 upregulates c-MYC by triggering the integrin β1/FAK/β-catenin signalling axis

**Figure 6:**
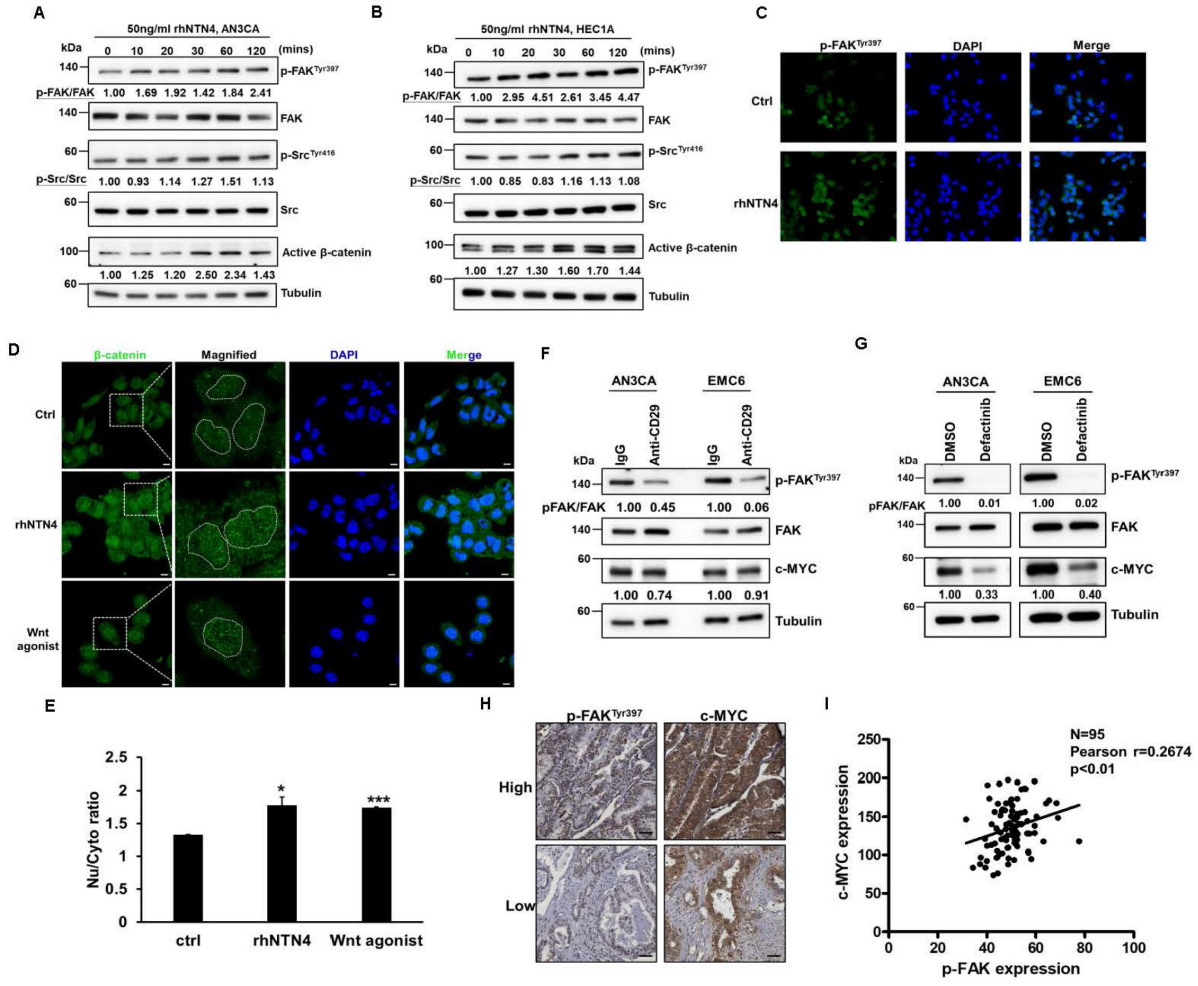
** Secreted Netrin4 upregulates c-MYC by triggering integrin β1/FAK/β-catenin signalling axis.** (A, B) Serum-starved AN3CA (A) and HEC1A (B) cells were treated with 50 ng/ml rhNTN4 and harvested at the indicated time points. Activation of FAK, SRC, and β-catenin was analysed using Western blot. (C) AN3CA cells, treated with 50 ng/ml rhNTN4 for 1 hour, had their FAK phosphorylation at Tyr397 visualized using immunofluorescence staining. DAPI was utilized for nuclear counterstaining. (D, E) AN3CA cells were treated with 50 ng/ml rhNTN4 or 20 µM Wnt agonist for 16 hours. β-catenin expression was then visualized using immunofluorescence staining (D). Scale bars represented 100 µm in length. The dotted circles in the magnified images highlight the cell nuclei based on DAPI signals. ImageJ was used to quantify the nuclear/cytoplasmic intensities of β-catenin. The ratios of nuclear/cytoplasmic β-catenin, determined from two objective fields, are presented as mean ± SD (E). *, p< 0.05; **, p< 0.01 by Student's *t*-test when compared to untreated control cells (ctrl). (F) AN3CA and EMC6 cells, treated with control IgG or 1 µg/ml anti-CD29 for 16 hours, were assessed for FAK phosphorylation and c-MYC expression using Western blot. (G) AN3CA and EMC6 cells, treated with either 0.01% DMSO or 1 µM Defactinib for 16 hours, were analyzed for FAK phosphorylation and c-MYC expression via Western blot. (H, I) The protein expression of p-FAK^Tyr397^ and c-MYC in EC tissues was visualized using IHC staining on tissue microarray slides. Representative images showcasing high/low staining intensities of p-FAK^Tyr397^ and c-MYC are provided in (H). Their correlation was then evaluated using Pearson's correlation analysis (I).

Focal adhesin kinase (FAK), a cytoplasmic kinase, functions as the crucial mediator of integrin signaling 41. FAK activation by integrin facilitates autophosphorylation at Tyr397, leading to the recruitment and activation of SRC kinase 42. As shown in Figs. 6A-C, secreted NTN4 can activate the downstream mediators (FAK and SRC) of integrin signaling in EC cells. FAK was reported to regulate the Wnt/β-catenin signaling pathway in breast and lung cancer 43. Moreover, a previous study indicated that nuclear β-catenin stabilization maintained CSC activity in EC 44. The findings of the present study showed that secreted NTN4 can increase the level of the active form (nuclear) of β-catenin (Figs. 6A and B) and the relative intensity of nuclear β-catenin staining (Figs. 6D and E) in EC cells. Nuclear accumulation of β-catenin activated the expression of target genes, including one pluripotent transcription factor, c-MYC 45. In addition, integrin β1 regulated the expression of c-MYC via activation of the FAK/SRC signaling axis in mammary epithelial cells 46. To investigate whether secreted NTN4 upregulates c-MYC by initiating the integrin-dependent FAK signaling pathway, EC cells were treated with an integrin β1 blockade antibody or defactinib, a small-molecule FAK inhibitor. Following these treatments, there was a notable decrease in the expression of p-FAK^Tyr397^ (Figs. 6F and G). Concurrently, c-MYC expression was diminished with defactinib treatment (Fig. 6G). Moreover, a positive correlation was found between the expression levels of p-FAK^Tyr397^ and c-MYC in EC specimens (Figs. 6H and I). Taken together, these results showed that secreted NTN4 can activate the integrin β1/FAK/β-catenin signaling axis and subsequently upregulate c-MYC in EC cells.

### EXOSC5 promotes cancer stemness through the MYC pathway

**Figure 7:**
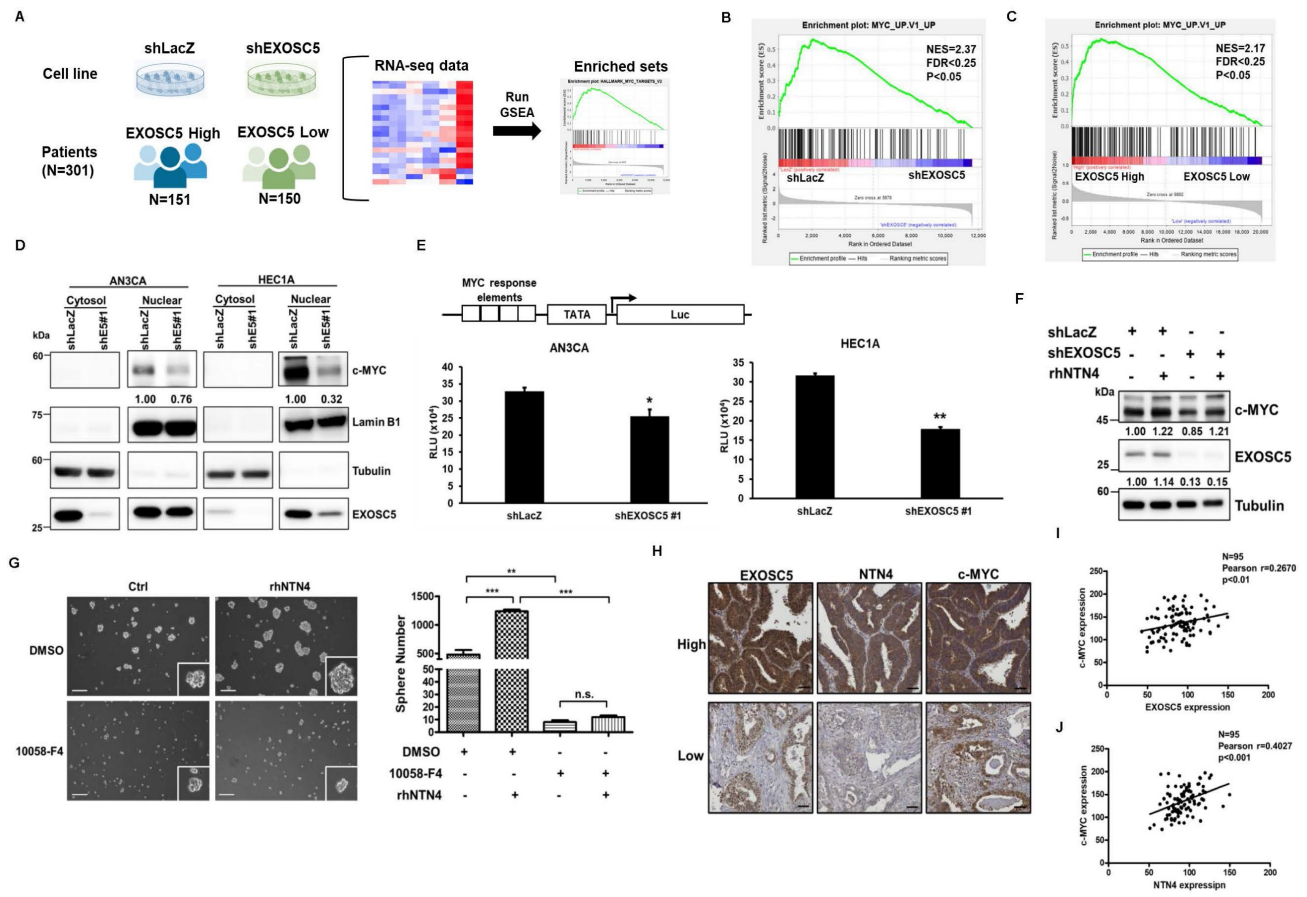
** EXOSC5 upregulates MYC pathway in ECs.** (A to C) The schematic diagram illustrating GSEA analytical procedures for the RNA-Seq data from EC cell lines post-EXOSC5 knockdown and EC specimens from the TCGA, categorized based on the median expression level of EXOSC5, is shown in (A). The GSEA analysis of the MYC pathway in EXOSC5-depleted versus control (shLacZ) HEC1A cells is presented in (B), while the GSEA analysis of the MYC pathway in the TCGA ESCA dataset is shown in (C). (D) From EXOSC5-knockdown AN3CA or HEC1A cells (shE5#1), cytosolic and nuclear fractions were extracted, and the expressions of EXOSC5 and c-MYC were probed via Western blot. (E) In both EXOSC5-depleted (shEXOSC5#1) and control (shLacZ) AN3CA or HEC1A cells, the transcriptional activity of c-MYC was assessed using a luciferase-based reporter assay. Data are denoted as mean ± SD. *, p< 0.05; **, p< 0.01 based on Student's *t*-test. (F) EXOSC5-depleted (shEXOSC5) and control (shLacZ) AN3CA cells were exposed to 50 ng/ml rhNTN4 for 48 hours, after which the expressions of EXOSC5 and c-MYC were evaluated using Western blot. (G) EMC6 cells were treated with 50 ng/ml rhNTN4 either alone or in combination with 10058-F4 (concentration: 100 µM). Subsequently, CSC activity was gauged using a tumorsphere assay. On the left panel, representative images of tumorspheres are shown, while the right panel displays quantification results, expressed as mean ± SD. **, p< 0.01; ***, p< 0.001; n.s., not significant, as determined by Tukey's Multiple Comparison Test. Scale bars represented 100 µm in length. (H, I, J) Protein expressions of EXOSC5, NTN4, and c-MYC in EC tissues were visualized via IHC staining on tissue microarray slides. Representative images highlighting the high/low staining intensities of each protein are provided in (H). Scale bars indicated a length of 50μm. Pearson's correlation analysis was employed to deduce the relationships between EXOSC5 and c-MYC (I) and between NTN4 and c-MYC (J).

Gene set enrichment analysis (GSEA) was utilized to examine the RNA-seq data from the EXOSC5-depleted HEC1A cells and patients with EC of TCGA to evaluate the potential pathways of EXOSC5 involved in EC-CSCs (Fig. 7A). The data revealed that the c-MYC target gene signature was significantly enriched in the EXOSC5-high group of EC cell lines or clinical samples (Figs. 7B and 7C). Moreover, the expression of nuclear c-MYC significantly decreased in EXOSC5-depleted EC cells (Fig. 7D). Dual luciferase reporter assay further showed that EXOSC5 upregulated the transcriptional activity of c-MYC in EC cells (Fig. 7E). EXOSC5-depleted and control EC cells were treated with rhNTN4 to study whether EXOSC5 upregulates the MYC pathway by regulating the secretion of NTN4. The results showed that rhNTN4 can restore c-MYC expression in EXOSC5 knockdown EC cells (Fig. 7F). To probe whether secreted NTN4 bolsters CSC activity through the activation of the c-MYC pathway, EC cells were administered with 10058-F4, a c-Myc-Max dimerization inhibitor, with or without subsequent rhNTN4 treatment. The results showed that 10058-F4 treatment can significantly decrease the CSC activity of rhNTN4-treated EC cells (Fig. 7G). A direct relationship was discerned between the expression levels of EXOSC5/c-MYC and NTN4/c-MYC in EC samples (Figs. 7H-7J). Collectively, these findings suggest that EXOSC5 promotes CSC activity through NTN4-induced MYC activation.

## Discussion

The RNA exosome activity of EXOSC members has been documented to play a role in tumor biology. Zhang et al. reported that EXOSC2 might regulate tumor angiogenesis in esophageal cancer by accelerating the degradation of IFI27 mRNA 47. Similarly, EXOSC4 has been shown to destabilize SESN2 mRNA, influencing the proliferation of pancreatic cancer cells 48. However, the present study showed that the number of upregulated mRNAs was limited in EXOSC5-knockdown EC cells (Fig. 3A). This suggests that the mechanism by which EXOSC5 regulates CSC maintenance in EC might be independent of RNA exosome activity. In other organisms, such as Caenorhabditis elegans, the EXOSC5 homolog, known as CRN-5, plays a role in the structural or catalytic component of the DNA cleavage complex during apoptosis 49. Moreover, EXOSC5 expression has been linked to an increase in cyclin D1 and a decrease in p21/p27 expression, which promotes cell proliferation through the regulation of the AKT/STAT3 pathway in gastric cancer 50. This STAT3-activating function of EXOSC5, which promotes cell proliferation, has also been observed in hepatocellular carcinoma 51. These findings lend further support to the idea of an RNA exosome-independent function for EXOSC5. Our current study underscores the crucial role of EXOSC5 in EC progression. Elevated expression of EXOSC5 correlates with a high histological grade and unfavorable prognosis in EC patients. Additionally, our findings suggest that NTN4, a downstream target of EXOSC5, might be a key regulator of EC-CSCs. We've unveiled that the secretion of NTN4, upon binding to integrin β1, activates the FAK/SRC/β-catenin signaling axis, leading to the enhancement of the MYC pathway.

NTN4 is involved in numerous biological processes, including tissue morphogenesis 52, angiogenesis 53, lymphangiogenesis 54, and tumorigenesis 55. In addition, a recent study indicated that NTN4 may support the formation of TME in breast cancer 56. However, most of these studies focused on the paracrine effects of NTN4 on tumor stromal cells, including fibroblasts, macrophages, lymphocytes, and endothelial cells. In the present study, the autocrine effect of NTN4 in regulating EC progression was investigated. The results identified secreted NTN4 as the ligand for integrin β1 and elucidated the downstream molecular mechanisms of EC-CSCs. Similar findings were observed in glioblastoma cells, where secreted NTN4 regulates cell proliferation via integrin β4 signaling 33. Therefore, NTN4 may serve as a regulator of integrin signaling in cancer progression.

Integrin β1 has been implicated in cancer progression, metastasis, and therapy resistance in numerous cancer types 38. Elevated levels of integrin β1 activate multiple integrin-dependent and cancer-related pathways, including FAK, ERK/MAPK, SRC, and AKT 18. Several studies showed that integrin β1 enhances cancer stemness in oral, lung, and colon cancer 38. The present study demonstrated that integrin β1 is an enriched marker of cancer stemness and a potential target for EC-CSCs (Fig. 5). In preclinical models, blockade of integrin β1 presented promising efficacy in reducing tumor burden. For example, volociximab, an α5β1-blockade antibody, was reported to impair angiogenesis and tumor growth in ovarian cancer xenograft models 57. Inhibition of FAK, a signal transducer for integrins with small-molecule inhibitors, such as defactinib (also known as VS-6063), showed promising treatment efficacy for patients with advanced non-small cell lung cancer in a phase II study 58. Additionally, the combination of defactinib, pembrolizumab, and gemcitabine demonstrated promising efficacy in a phase I study for pancreatic cancer treatment 59. In the present study, treatment with defactinib can impair the MYC pathway through inhibition of FAK activation in EC cells. These results highlighted the therapeutic potential of targeting FAK in EC-CSC treatment.

## Conclusions

The present investigation distinctly emphasizes the pivotal role of EXOSC5 in the intricate progression of EC. A notable association emerges between heightened EXOSC5 expression and elevated histological grading and adverse patient prognoses. Intriguingly, our research alludes to NTN4, modulated downstream of EXOSC5, as a possibly indispensable regulator of EC-CSC maintenance. Mechanistically, we discovered that NTN4 secretion, after its affiliation with integrin β1, instigates the FAK/SRC/β-catenin signaling cascade, thereby amplifying the MYC pathway. Such revelations bolster the potential of EXOSC5 and NTN4 as diagnostic targets of EC prognosis. Moreover, the therapeutic horizon for EC might be expanded by targeting the integrin β1/FAK/MYC signaling axis using small molecular antagonists such as defactinib and 10058-F4 or through a selective antibody blockade of integrin β1, given their pronounced effect in curtailing EC's cancer stemness attributes. In conclusion, the present study elucidates the molecular mechanism by which EXOSC5 positively regulates EC-CSC function, potentially providing EXOSC5-/NTN4-centered diagnostic and therapeutic approaches in EC, encompassing multi-faceted target points inclusive of EC-CSCs.

## Supplementary Material

Supplementary figures.

## Abbreviations

EC: Endometrial carcinoma
CSCs: Cancer stem cells
EXOSC5: Exosome component 5
NTN4: Netrin4
FAK: focal adhesion kinase
TCGA: The Cancer Genome Atlas
CM: conditioned medium
GO: gene ontology
GSEA: gene set enrichment analysis

## References

[1] Cramer DW (2012). The epidemiology of endometrial and ovarian cancer. Hematol Oncol Clin North Am.

[2] Chen JY, Kuo SJ, Liaw YP, Avital I, Stojadinovic A, Man YG (2014). Endometrial cancer incidence in breast cancer patients correlating with age and duration of tamoxifen use: a population based study. J Cancer.

[3] Murali R, Soslow RA, Weigelt B (2014). Classification of endometrial carcinoma: more than two types. Lancet Oncol.

[4] Sarmadi S, Izadi-Mood N, Sotoudeh K, Tavangar SM (2009). Altered PTEN expression; a diagnostic marker for differentiating normal, hyperplastic and neoplastic endometrium. Diagn Pathol.

[5] Slomovitz BM, Coleman RL (2012). The PI3K/AKT/mTOR Pathway as a Therapeutic Target in Endometrial Cancer. Clin Cancer Res.

[6] Zheng W, Xiang L, Fadare O, Kong B (2011). A proposed model for endometrial serous carcinogenesis. Am J Surg Pathol.

[7] Lee W-L, Yen M-S, Chao K-C, Yuan C-C, Ng H-T, Chao H-T (2014). Hormone therapy for patients with advanced or recurrent endometrial cancer. J Chin Med Assoc.

[8] Giannone G, Attademo L, Scotto G, Genta S, Ghisoni E, Tuninetti V (2019). Endometrial Cancer Stem Cells: Role, Characterization and Therapeutic Implications. Cancers (Basel).

[9] Vermeulen L, de Sousa e Melo F, Richel DJ, Medema JP (2012). The developing cancer stem-cell model: clinical challenges and opportunities. The Lancet Oncology.

[10] Visvader JE, Lindeman GJ (2012). Cancer stem cells: current status and evolving complexities. Cell Stem Cell.

[11] Teeuwssen M, Fodde R (2019). Wnt Signaling in Ovarian Cancer Stemness, EMT, and Therapy Resistance. Journal of Clinical Medicine.

[12] Lizárraga-Verdugo E, Avendaño-Félix M, Bermúdez M, Ramos-Payán R, Pérez-Plasencia C, Aguilar-Medina M (2020). Cancer Stem Cells and Its Role in Angiogenesis and Vasculogenic Mimicry in Gastrointestinal Cancers. Front Oncol.

[13] Lei MML, Lee TKW (2021). Cancer Stem Cells: Emerging Key Players in Immune Evasion of Cancers. Frontiers in cell and developmental biology.

[14] Lin H, Wang B, Yu J, Wang J, Li Q, Cao B (2018). Protein arginine methyltransferase 8 gene enhances the colon cancer stem cell (CSC) function by upregulating the pluripotency transcription factor. J Cancer.

[15] Kang L, Mao J, Tao Y, Song B, Ma W, Lu Y (2015). MicroRNA-34a suppresses the breast cancer stem cell-like characteristics by downregulating Notch1 pathway. Cancer Sci.

[16] Ji C, Yang L, Yi W, Xiang D, Wang Y, Zhou Z (2018). Capillary morphogenesis gene 2 maintains gastric cancer stem-like cell phenotype by activating a Wnt/β-catenin pathway. Oncogene.

[17] Deng J, Bai X, Feng X, Ni J, Beretov J, Graham P (2019). Inhibition of PI3K/Akt/mTOR signaling pathway alleviates ovarian cancer chemoresistance through reversing epithelial-mesenchymal transition and decreasing cancer stem cell marker expression. BMC Cancer.

[18] Cooper J, Giancotti FG (2019). Integrin Signaling in Cancer: Mechanotransduction, Stemness, Epithelial Plasticity, and Therapeutic Resistance. Cancer Cell.

[19] Park CY, Tseng D, Weissman IL (2009). Cancer stem cell-directed therapies: recent data from the laboratory and clinic. Mol Ther.

[20] Mori Y, Yamawaki K, Ishiguro T, Yoshihara K, Ueda H, Sato A (2019). ALDH-Dependent Glycolytic Activation Mediates Stemness and Paclitaxel Resistance in Patient-Derived Spheroid Models of Uterine Endometrial Cancer. Stem cell reports.

[21] Kim M, Lee S, Park WH, Suh DH, Kim K, Kim YB (2018). Silencing Bmi1 expression suppresses cancer stemness and enhances chemosensitivity in endometrial cancer cells. Biomed Pharmacother.

[22] Zhou X, Zhou YP, Huang GR, Gong BL, Yang B, Zhang DX (2011). Expression of the stem cell marker, Nanog, in human endometrial adenocarcinoma. Int J Gynecol Pathol.

[23] Morton DJ, Kuiper EG, Jones SK, Leung SW, Corbett AH, Fasken MB (2018). The RNA exosome and RNA exosome-linked disease. RNA.

[24] Guo X, Ma J, Sun J, Gao G (2007). The zinc-finger antiviral protein recruits the RNA processing exosome to degrade the target mRNA. Proc Natl Acad Sci U S A.

[25] Puno MR, Weick EM, Das M, Lima CD (2019). SnapShot: The RNA Exosome. Cell.

[26] Goodarzi H, Nguyen HCB, Zhang S, Dill BD, Molina H, Tavazoie SF (2016). Modulated Expression of Specific tRNAs Drives Gene Expression and Cancer Progression. Cell.

[27] Stefanska B, Cheishvili D, Suderman M, Arakelian A, Huang J, Hallett M (2014). Genome-wide study of hypomethylated and induced genes in patients with liver cancer unravels novel anticancer targets. Clin Cancer Res.

[28] Yang X-F, Wu CJ, Chen L, Alyea EP, Canning C, Kantoff P (2002). CML28 Is a Broadly Immunogenic Antigen, Which Is Overexpressed in Tumor Cells. Cancer Res.

[29] Basu U, Meng FL, Keim C, Grinstein V, Pefanis E, Eccleston J (2011). The RNA exosome targets the AID cytidine deaminase to both strands of transcribed duplex DNA substrates. Cell.

[30] Pan H, Pan J, Song S, Ji L, Lv H, Yang Z (2019). EXOSC5 as a Novel Prognostic Marker Promotes Proliferation of Colorectal Cancer via Activating the ERK and AKT Pathways. Front Oncol.

[31] Elbasateeny SS, Salem AA, Abdelsalam WA, Salem RA (2016). Immunohistochemical expression of cancer stem cell related markers CD44 and CD133 in endometrial cancer. Pathol Res Pract.

[32] Bruikman CS, Zhang H, Kemper AM, van Gils JM (2019). Netrin Family: Role for Protein Isoforms in Cancer. Journal of Nucleic Acids.

[33] Hu Y, Ylivinkka I, Chen P, Li L, Hautaniemi S, Nyman TA (2012). Netrin-4 Promotes Glioblastoma Cell Proliferation through Integrin β4 Signaling. Neoplasia.

[34] Lv B, Song C, Wu L, Zhang Q, Hou D, Chen P (2015). Netrin-4 as a biomarker promotes cell proliferation and invasion in gastric cancer. Oncotarget.

[35] Staquicini FI, Dias-Neto E, Li J, Snyder EY, Sidman RL, Pasqualini R (2009). Discovery of a functional protein complex of netrin-4, laminin γ1 chain, and integrin α6β1 in mouse neural stem cells. Proceedings of the National Academy of Sciences.

[36] Larrieu-Lahargue F, Welm AL, Thomas KR, Li DY (2011). Netrin-4 Activates Endothelial Integrin Integrin a6b1. Circ Res.

[37] Su C-y, Li J-q, Zhang L-l, Wang H, Wang F-h, Tao Y-w (2020). The Biological Functions and Clinical Applications of Integrins in Cancers. Front Pharmacol.

[38] Xiong J, Yan L, Zou C, Wang K, Chen M, Xu B (2021). Integrins regulate stemness in solid tumor: an emerging therapeutic target. J Hematol Oncol.

[39] Wu X, Cai J, Zuo Z, Li J (2019). Collagen facilitates the colorectal cancer stemness and metastasis through an integrin/PI3K/AKT/Snail signaling pathway. Biomed Pharmacother.

[40] Lin HC, Wu CL, Chen YL, Huang JS, Wong TY, Yuan K (2014). High-level β1-integrin expression in a subpopulation of highly tumorigenic oral cancer cells. Clin Oral Investig.

[41] Guan JL (2010). Integrin signaling through FAK in the regulation of mammary stem cells and breast cancer. IUBMB life.

[42] Bolós V, Gasent JM, López-Tarruella S, Grande E (2010). The dual kinase complex FAK-Src as a promising therapeutic target in cancer. Onco Targets Ther.

[43] Wörthmüller J, Rüegg C (2020). The Crosstalk between FAK and Wnt Signaling Pathways in Cancer and Its Therapeutic Implication. Int J Mol Sci.

[44] Yokoi A, Minami M, Hashimura M, Oguri Y, Matsumoto T, Hasegawa Y (2022). PTEN overexpression and nuclear β-catenin stabilization promote morular differentiation through induction of epithelial-mesenchymal transition and cancer stem cell-like properties in endometrial carcinoma. Cell Communication and Signaling.

[45] Jeong W-J, Ro EJ, Choi K-Y (2018). Interaction between Wnt/β-catenin and RAS-ERK pathways and an anti-cancer strategy via degradations of β-catenin and RAS by targeting the Wnt/β-catenin pathway. npj Precision Oncology.

[46] Benaud CM, Dickson RB (2001). Regulation of the expression of c-Myc by beta1 integrins in epithelial cells. Oncogene.

[47] Zhang Y, Chen C, Liu Z, Guo H, Lu W, Hu W (2022). PABPC1-induced stabilization of IFI27 mRNA promotes angiogenesis and malignant progression in esophageal squamous cell carcinoma through exosomal miRNA-21-5p. J Exp Clin Cancer Res.

[48] Taniue K, Tanu T, Shimoura Y, Mitsutomi S, Han H, Kakisaka R (2022). RNA Exosome Component EXOSC4 Amplified in Multiple Cancer Types Is Required for the Cancer Cell Survival. Int J Mol Sci.

[49] Yang CC, Wang YT, Hsiao YY, Doudeva LG, Kuo PH, Chow SY (2010). Structural and biochemical characterization of CRN-5 and Rrp46: an exosome component participating in apoptotic DNA degradation. RNA.

[50] Chen X, Huang Y, Liu J, Lin W, Chen C, Chen Y (2022). EXOSC5 promotes proliferation of gastric cancer through regulating AKT/STAT3 signaling pathways. J Cancer.

[51] Zhang Y, Yang X, Hu Y, Huang X (2022). Integrated Bioinformatic Investigation of EXOSCs in Hepatocellular Carcinoma Followed by the Preliminary Validation of EXOSC5 in Cell Proliferation. Int J Mol Sci.

[52] Liu Y, Stein E, Oliver T, Li Y, Brunken WJ, Koch M (2004). Novel role for Netrins in regulating epithelial behavior during lung branching morphogenesis. Curr Biol.

[53] Lambert E, Coissieux MM, Laudet V, Mehlen P (2012). Netrin-4 acts as a pro-angiogenic factor during zebrafish development. J Biol Chem.

[54] Larrieu-Lahargue F, Welm AL, Thomas KR, Li DY (2010). Netrin-4 induces lymphangiogenesis *in vivo*. Blood.

[55] Eveno C, Broqueres-You D, Feron JG, Rampanou A, Tijeras-Raballand A, Ropert S (2011). Netrin-4 delays colorectal cancer carcinomatosis by inhibiting tumor angiogenesis. Am J Pathol.

[56] Yi L, Lei Y, Yuan F, Tian C, Chai J, Gu M (2022). NTN4 as a prognostic marker and a hallmark for immune infiltration in breast cancer. Sci Rep.

[57] Bhaskar V, Zhang D, Fox M, Seto P, Wong MH, Wales PE (2007). A function blocking anti-mouse integrin alpha5beta1 antibody inhibits angiogenesis and impedes tumor growth *in vivo*. J Transl Med.

[58] Gerber DE, Camidge DR, Morgensztern D, Cetnar J, Kelly RJ, Ramalingam SS (2020). Phase 2 study of the focal adhesion kinase inhibitor defactinib (VS-6063) in previously treated advanced KRAS mutant non-small cell lung cancer. Lung Cancer.

[59] Wang-Gillam A, Lim K-H, McWilliams R, Suresh R, Lockhart AC, Brown A (2022). Defactinib, Pembrolizumab, and Gemcitabine in Patients with Advanced Treatment Refractory Pancreatic Cancer: A Phase I Dose Escalation and Expansion Study. Clin Cancer Res.

