# Supplementary material for: EXOSC5 maintains cancer stem cell activity in endometrial cancer by regulating the NTN4/integrin β1 signalling axis
Source: Int J Biol Sci. 2024 Jan 1;20(1):265–79. doi: 10.7150/ijbs.86275 (PMC10750274; doi:10.7150/ijbs.86275)
Supplement: Supplementary file 1 — Supplementary figures. [file ijbsv20p0265s1.pdf]

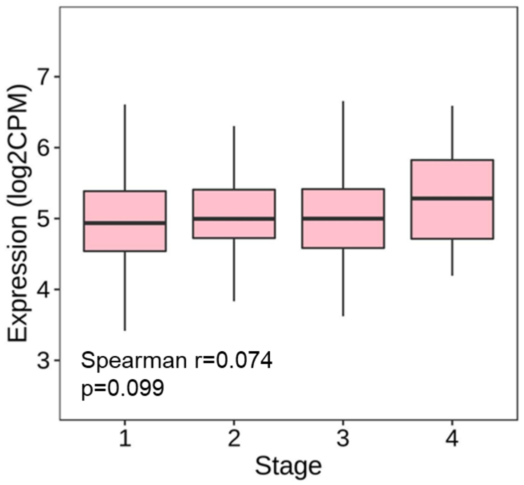

Figure S1. EXOSC5 is not significantly associated with tumor stage in EC dataset of TCGA database. Spearman correlation analysis of EXOSC5 and tumor stage in EC specimens was plotted by TISIDB webtool.

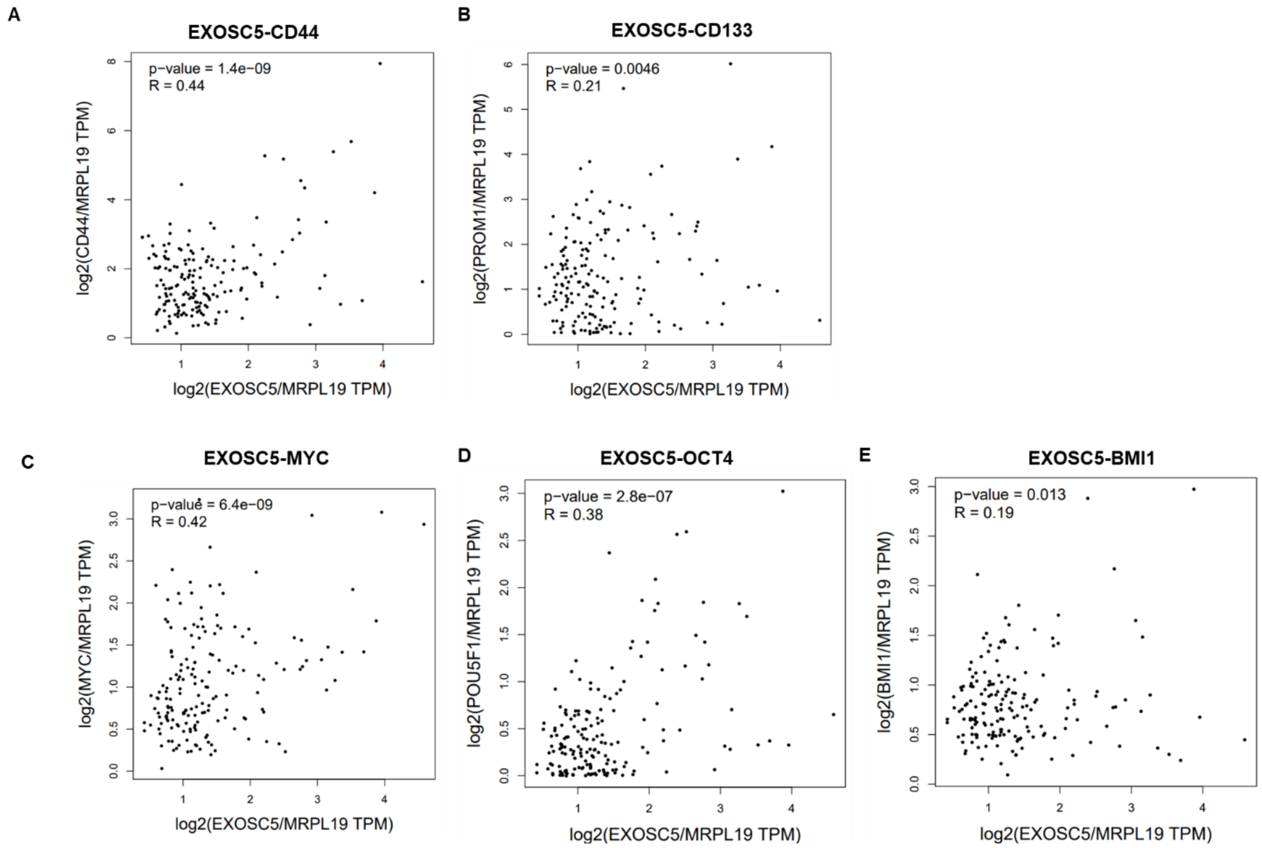

Figure S2. EXOSC5 positively corelates with various genes related to cancer stemness in EC dataset of TCGA database. (A-E) Pearson's correlation analysis of EXOSC5 and CD44 (A), CD133 (B), MYC (C), OCT4 (D), BMI1 (E) in EC specimens were plotted by GEPIA2 webtool (Tang et al., 2017).

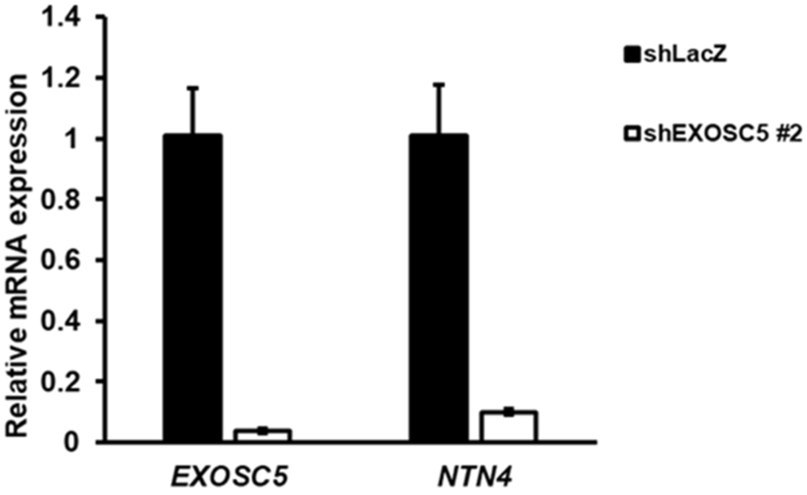

Figure S3. EXOSC5 positively regulates NTN4 mRNA expression in EC cells. Total RNA was extracted from EXOSC5-depletion (shEXOSC5) and control (shLacZ) HEC1A cells and the mRNA expressions of EXOSC5 and NTN4 were determined by qRT-PCR. Data are represented as mean ± SD. **, $p>0.01$, student's t test.

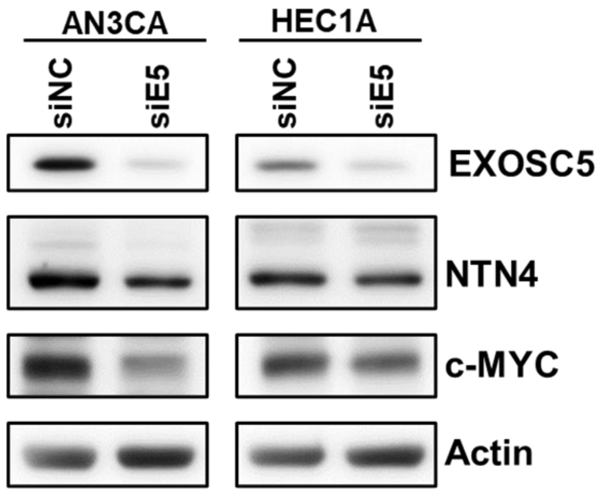

Figure S4. EXOSC5 knockdown would downregulate NTN4 and c-MYC expression. AN3CA and HEC1A cells were transfected with specific siRNA followed by 72 hours and the protein expressions of EXOSC5, NTN4, and c-MYC were determined by Western blot. siNC, control siRNA(sc-37007); siE5, EXOSC5 siRNA (sc-97360).

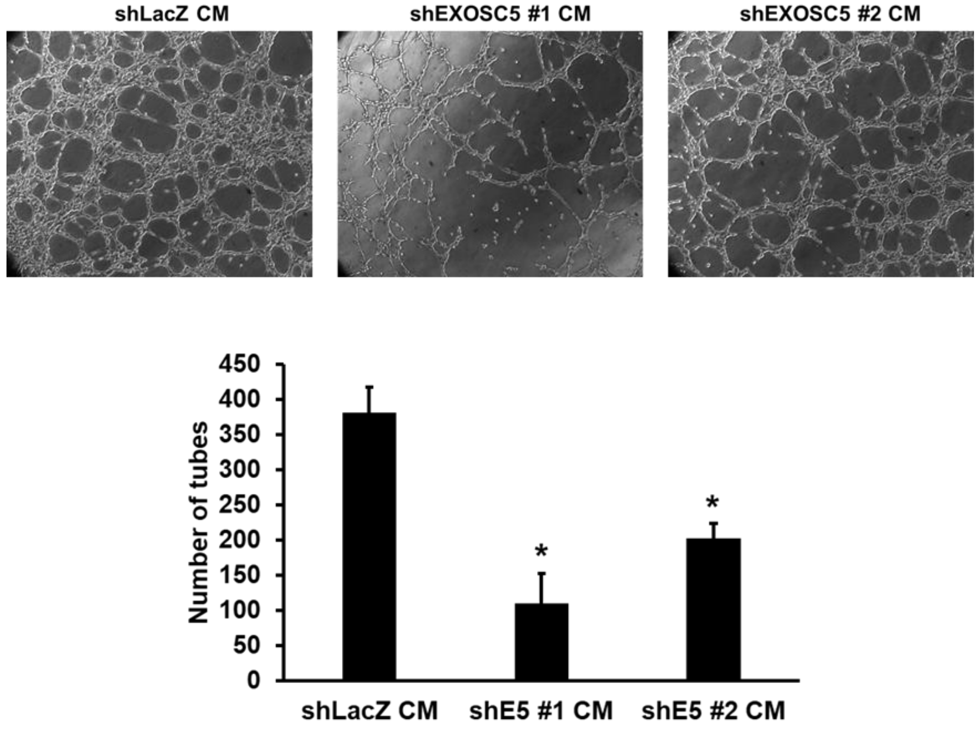

Figure S5. Condition medium (CM) from EXOSC5-depletion AN3CA cells could decrease tube formation capability. Wells of μ-Slide Angiogenesis (ibidi, 81506) were pre-coated with 4mg/ml Matrigel for 1hr at 37°C. HMEC-1 (1x10^4 cells/well) were then seeded in the presence of condition medium derived from EXOSC5-depletion or control EC cells. The tubular formation was observed and photographed by microscope after incubation at 37 °C for 4-8 hr. The quantification determined the number branch points per field. Data are represented as mean ± SD. *, p>0.05, student's t test.

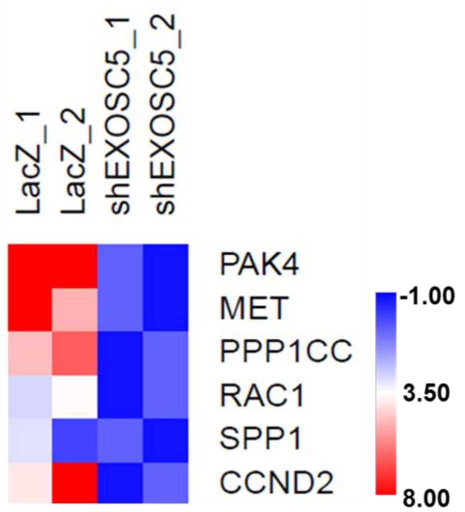

ID	Description	P value
hsa04510	Focal adhesion	0.032

Figure S6. EXOSC5 positively regulates focal adhesion pathway in EC cells. KEGG pathway analysis of EXOSC5-depletion HEC1A cells and shLacZ-transduced control cells.

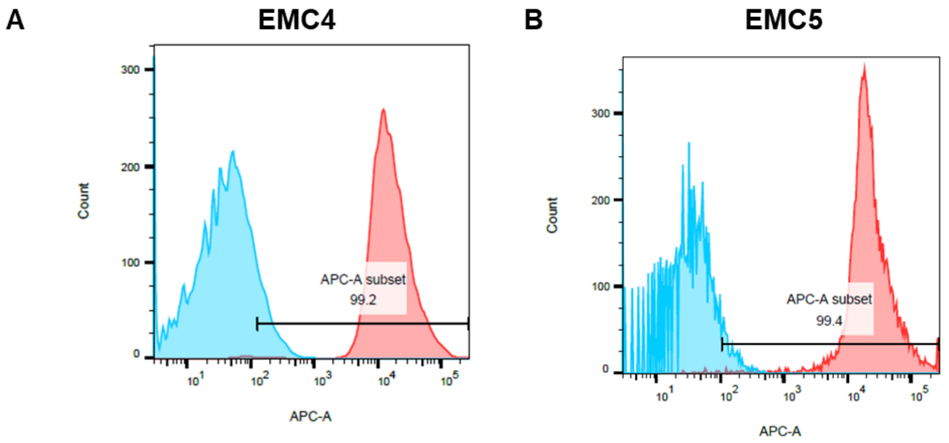

Figure S7. Primary EMC4 and EMC5 possessed high endogenous integrin β1 expression. Cells stained with APC-conjugated anti-CD29 antibody at room temperature for 30 min. The fluorescence signals of APC were captured with the FACSCantoII flow cytometer (BD Biosciences). The positive percentage of CD29 expression were analyzed with FlowJo software.
